# Impact of Radiation Dose on Survival for Esophageal Squamous Cell Carcinoma Treated With Neoadjuvant Chemoradiotherapy

**DOI:** 10.3389/fonc.2020.01431

**Published:** 2020-08-20

**Authors:** Yang Yang, Xiaofang Xu, Xia Zhou, Wuan Bao, Danhong Zhang, Feiying Gu, Xianghui Du, Qixun Chen, Guoqin Qiu

**Affiliations:** ^1^Department of Thoracic Radiotherapy, Cancer Hospital of the University of Chinese Academy of Sciences (Zhejiang Cancer Hospital), Hangzhou, China; ^2^Institute of Cancer and Basic Medicine (IBMC), Chinese Academy of Sciences, Hangzhou, China; ^3^Department of Thoracic Surgery, Cancer Hospital of the University of Chinese Academy of Sciences (Zhejiang Cancer Hospital), Hangzhou, China

**Keywords:** esophageal cancer, neoadjuvant chemoradiotherapy, surgery, radiation dose, trimodality cancer treatment

## Abstract

**Purpose:** Radiation dose used in the neoadjuvant chemoradiotherapy (NCRT) for patients with locally advanced esophageal squamous cell carcinoma (ESCC) varies in different trials and clinical practice.

**Methods and Materials:** Data from patients diagnosed with ESCC receiving NCRT followed by esophagectomy were retrospectively collected from February 2013 to December 2017. Lower dose (LD) radiotherapy was defined as ≤45 Gy, and >45 Gy was considered as higher dose (HD). Survival rates were calculated by the Kaplan–Meier method and compared with long-rank test. Multivariate Cox regression analyses were performed to identify variables associated with survival.

**Results:** A total of 118 patients treated with NCRT were included in our analysis: 62 patients received LD radiotherapy, and 56 patients received HD radiotherapy. The median follow-up time was 24.3 months (0.67–65.3 m). Two-years overall survival (OS) rates were 75.0 and 79.0% in HD and LD group, respectively (*P* = 0.360), and complete pathological remission (pCR) rates in two groups were 42.9 and 30.6%, respectively (*P* = 0.17). The incidences of toxic effects including post-operative complications were not significantly different between two groups. Multivariate analysis showed that tumor T stage, M1a disease, smoking history, and pCR rate were significantly associated with OS.

**Conclusions:** In ESCC patients treated with NCRT followed by surgery, higher radiation dose was not significantly associated with a higher pCR rate and longer survival. Lower radiation dose might be a preferable time-dose fraction scheme. Our finding needs to be further validated by randomized trials.

## Introduction

Esophageal cancer is one of the most common cancers globally, and esophageal squamous cell carcinoma (ESCC) is the major subtype in Eastern Asia, especially in China, accounting for about 90% of newly diagnosed esophageal cancers each year (1). More than half of ESCC patients are diagnosed at locally advanced stage, and outcomes of patients receiving surgical resection alone are still unsatisfactory, with a 5-years survival rate of 20–36% (2). Therefore, multidisciplinary collaboration is vitally important for the success of ESCC treatment (3).

By pooling all previous trials together in the past decades, two meta-analyses systematically found a significant survival benefit from multimodality treatment in patients with esophageal carcinoma (4, 5). Then, the publication of the CROSS trial provides more evidence for NCRT before surgery, which has been established as the standard of care for patients with locally advanced esophageal cancer (6). Compared to esophageal adenocarcinoma, patients with ESCC tend to gain more benefits from NCRT, which was confirmed by a NEOCRTEC5010 study from China (7).

However, the scheme of NCRT itself still needs to be improved and optimized. Radiation doses used in the NCRT vary greatly in different trials and clinical practice, ranging from 20 Gy/10F, 41.4 Gy/23F, to 50.4 Gy/28F (6, 8, 9). Theoretically, dose escalation might increase local tumor control and pathological complete remission (pCR) rate. However, high radiation dose will also induce more severe side effects, especially post-operative complications, including anastomotic leakage, hemorrhea, pneumonia, and even death within 30 days after surgery, which might counterweigh the potential benefits of neoadjuvant therapy. The identification for optimal radiation dose in NCRT should balance both benefits and possible side effects, which remains to be further investigated.

To our knowledge, no prospective clinical trial has been designed to evaluate the effect of radiation dosage on the tumor response and survival of esophageal cancer in the context of NCRT up to now. To answer this question, we retrospectively collected and reviewed the ESCC patients in a single institution, to explore the influence of radiation dose on the survival of ESCC patients as well as the side effects. We hypothesized that higher radiation dose might increase pCR rate and possibly be associated with better survival.

## Methods and Materials

### Patient Selection

Retrospectively, patients with thoracic esophageal squamous carcinoma who received NCRT before surgery were reviewed at Zhejiang Cancer Hospital between January 2013 and July 2017. At first, 135 patients were searched from electronic medical database, among which 17 patients were excluded because of (1) other primary cancer history or a second malignancy (*n* = 7); (2) the interval between the completion of CRT to esophagectomy was more than 3 months (*n* = 4); (3) loss to follow-up (*n* = 6). The following information of patients was collected: age, gender, performance status, smoking and drinking status, clinical stage, radiation dose, chemotherapy, and follow-up results. For clinical TNM classification, the 6th version of the American Joint Committee on Cancer guidelines (6th AJCC, 2009) was employed.

### Treatment

Initially, all patients underwent pretreatment examinations including barium swallow, neck, thorax, and abdomen plain and contrast-enhanced computed tomography (CT), cervical and supraclavicular ultrasonography, and esophagogastroduodenoscopy, while fuorodeoxyglucose positron emission tomography (PET-CT), ultrasound endoscopy (EUS), radionuclide bone imaging, and brain MRI were optional. If necessary, bronchoscopy was conducted to exclude tumor invasion into the trachea or bronchial tree.

All the therapeutic approaches were in accordance with the NCCN or Chinese cancer guidelines and regulations. Radiation therapy was delivered using intensity-modulated RT (IMRT), with 6–8 MV X-ray irradiation. A total dose of 40–50.4 Gy was given in 1.8–2.0 Gy/fraction, 5 days per week. Different time-dose fraction schemes were employed in different treatment groups, including 39.6 Gy/22F (5 patients), 40 Gy/20F (3 patients), 41.4 Gy/23F (34 patients), 45 Gy/25F (18 patients), 46 Gy/23F (5 patients), 50 Gy/25F (4 patients), or 50.4 Gy/28F (49 patients). To be convenient, the median value 45 Gy was selected as a cutoff point in our analysis, which will also maintain balance of patient numbers between the two groups. A total dose >45 Gy was defined as high dose radiation (HD), and ≤45 Gy belonged to the group of low dose radiation (LD). The gross tumor volume (GTV) contained both the primary tumor and metastatic lymph nodes, determined by the results of barium swallow, endoscopy, contrast-enhanced CT, or PET-CT. The clinical target volume (CTV) was delineated by expanding the GTV by 3.0–4.0 cm at the proximal and distal margins and by 0.5–1.0 cm at the transversal margins. The planning target volume was defined as the CTV plus a margin of 0.5 cm, accounting for motion and setup variations.

During radiotherapy, paclitaxel (45 mg/m^2^) and carboplatin (AUC = 2) were administered weekly, or two cycles of PF regimen (DDP = 25 mg/m^2^ d1-3, 5-FU = 1,000 mg/m^2^ d1-3) were given every 3 weeks. Patients underwent surgery within 4–8 weeks after the completion of NCRT, Ivor-Lewis esophagectomy or McKeown esophagectomy with lymph node dissection was performed.

### Follow-Up and Outcomes

During NCRT, toxicity was assessed every week according to the National Cancer Institute Common Terminology Criteria for Adverse Events (version 3.0). Surgical complications were recorded within 1 month after resection. Then, all patients were followed every 3 months for the first 2 years, thereafter at 6-months intervals in the next 3 years. Physical examinations, chest CT, and barium scans were performed regularly during each follow-up, and ultrasonography and endoscopy or PET-CT and MRI were employed if necessary.

Overall survival (OS) was calculated from the beginning of NCRT until death from any cause or last follow-up. Disease-free survival (DFS) referred to the length of time from the start of treatment to the first documentation of recurrence, metastasis, or death. Local recurrence free survival (LRFS) and distant metastases-free survival (DMFS) were calculated from the start of treatment to the date of local-regional recurrence or distant metastasis, respectively.

### Statistical Analysis

For comparisons of baseline patient characteristics between the two groups, Mann-Whitney test was used for continuous variables whereas Pearson's chi-squared test was used for categorical data. The Kaplan–Meier method was used to construct the survival curves, and differences between two groups were compared by the log-rank test. Cox proportional hazards regression model was performed to identify potential prognostic factors for OS. All statistical analyses were two-sided with significance defined as *p* < 0.05, using the IBM SPSS software ver. 22.0 (IBM Corp., Armonk, NY).

## Results

### Patient Characteristics

Between January 2013 and July 2017, 118 patients who received NCRT followed by surgery were included in our study. Among them, 62 patients received ≤45 Gy of RT (low-dose group) and 56 patients received >45 Gy (high-dose group). The median follow-up time was 24.3 months (range 0.67–65.3) for all patients. Baseline characteristics of patients were shown in Table 1. Patients receiving high dose RT tended to be younger, and had less lesions in the lower thoracic region. No statistically significant differences were found between groups with respect to most variables, including gender, smoking, drinking, tumor length, clinical T stage, N stage, or M stage.

**Table 1:** Baseline patient, tumor, and treatment characteristics.

**Variables**	**RT dose ≤ 45 Gy (*N* = 62)**	**RT dose > 45 Gy (*N* = 56)**	***P*-value**
**Age**	60.1 years (47–77)	57.3 years (42–71)	**0.046**
**Gender**			0.44
Male	57 (51.4%)	54 (48.6%)	
Female	5 (71.4%)	2 (28.6%)	
**Smoking**			0.43
Yes	45 (50.6%)	44 (49.4%)	
No	17 (58.6%)	12 (41.4%)	
**Drinking**			0.11
Yes	46 (48.9%)	48 (51.1%)	
No	16 (66.7%)	8 (33.3%)	
**ECOG score**			0.32
0	17 (60.7%)	11 (39.3%)	
1	45 (50%)	45 (50%)	
**Clinical T stage**			0.76
2	12 (60%)	8 (40%)	
3	46 (51.1%)	44 (48.9%)	
4	4 (50%)	4 (50%)	
**Clinical N stage**			0.52
0	17 (58.6%)	12 (41.4%)	
1	45 (50.6%)	44 (49.4%)	
**Clinical M stage**			0.89
0	56 (52.3%)	51 (47.7%)	
1A	6 (54.5%)	5 (45.5%)	
**Clinical stage**			0.70
2a	12 (60.0%)	8 (40.0%)	
2b	8 (44.4%)	10 (55.6%)	
3	36 (52.2%)	33 (47.8%)	
4a	6 (54.5%)	5 (45.5%)	
**Location**			**0.032**
Upper	12 (46.2%)	14 (53.8%)	
Mid	36 (45.0%)	38 (55.0%)	
Lower	14 (77.8%)	4 (22.2%)	
**Tumor length**			0.062
≤ 5	9 (36.0%)	16 (64.0%)	
>5	53 (57.0%)	40 (43.0%)	
**Concurrent chemo**			0.14
≥4 cycles	28 (59.6%)	33 (40.0%)	
<4 cycles	34 (45.9%)	23 (54.1%)	
**Adjuvant chemo**			0.24
Yes	12 (42.9%)	16 (57.1%)	
No	50 (55.6%)	40 (44.4%)	

*The bold values are indicated as statistically significant*.

### Survival

**Figure 1:**
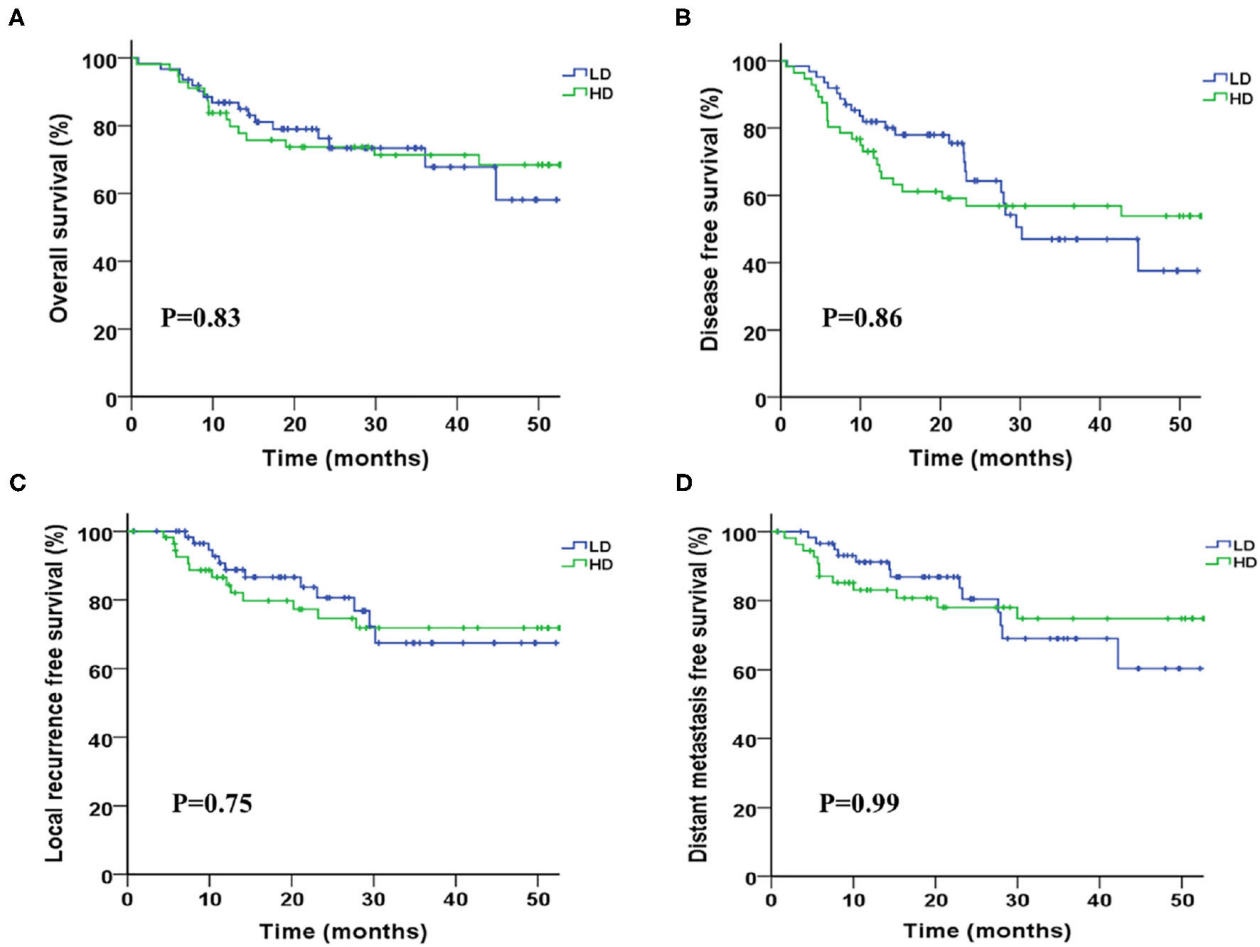
Kaplan-Meier survival curves between groups of higher and lower radiation dose. **(A)** Overall survival, **(B)** disease progression-free survival, **(C)** local progression-free survival, and **(D)** distant metastasis-free survival.

**Figure 2:**
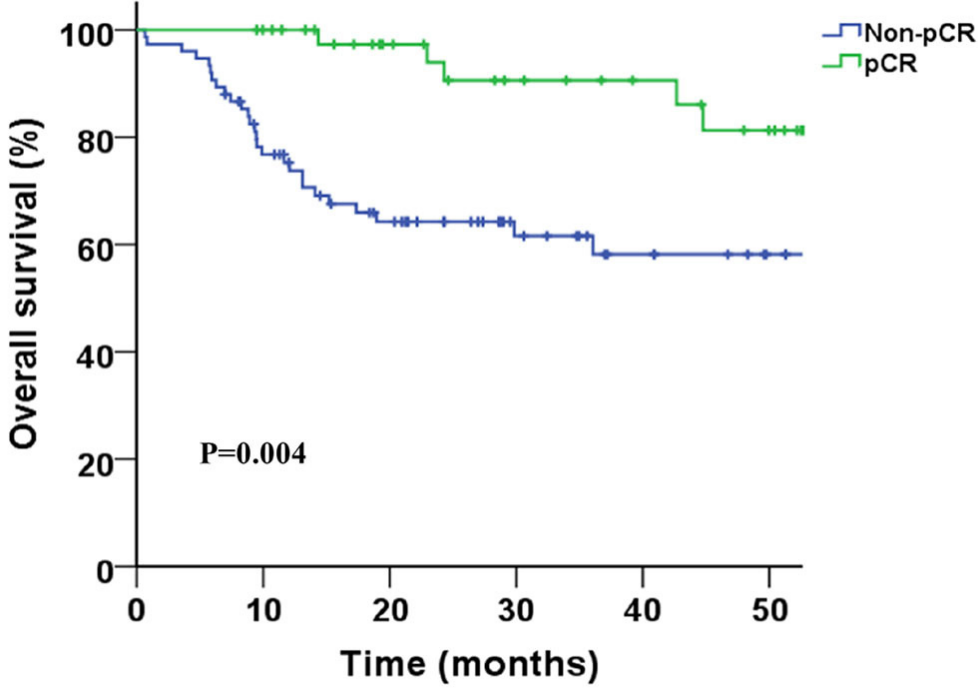
Kaplan-Meier survival curves for pathological complete response (pCR) vs. non-pCR patients.

Two-years overall survival (OS) rates were 75.0 and 79.0% in HD and LD group, respectively (*P* = 0.36), and the median OS was not achieved in either group until to last follow-up. Survival curves between the two groups were shown in Figure 1, and no significant differences were found regarding OS (*P* = 0.83), DFS (*P* = 0.86), LRFS (*P* = 0.75), and DMFS (*P* = 0.99). R0 resection rate in two groups were 93.5 and 94.6% in LD and HD group, respectively (*P* = 0.81). The complete pathological remission (pCR) rate seemed to be higher in the HD group than that in LD group (42.9 vs. 30.6%); however, the result was statistically non-significant (*P* = 0.17). Complete pathological response was significantly associated with better survival (HR = 0.35, 0.16–0.74, *p* < 0.01), as shown in Figure 2.

Univariate analysis revealed that T stage, M1a disease, and pCR were significant prognostic factors associated with OS. After multivariate Cox regression analysis, tumor T stage, M1a disease, smoking history, and pCR were found to be significantly associated with OS independently (Table 2).

**Table 2:** Univariate and multivariate Cox proportional hazards analysis of overall survival.

**Variables**	**Univariate**			**Multivariate**		
	**HR**	**95%CI**	***P*-value**	**HR**	**95%CI**	***P*-value**
**Age**						
≤ 60 years	Ref.					
>60 years	0.98	0.51–1.90	0.96			
**ECOG**						
0	Ref.					
1	0.93	0.44–1.96	0.84			
**T stage**						
2	Ref.			Ref.		
3	1.73	0.59–5.10	0.32	2.25	0.66–7.64	0.20
4	8.90	2.45–32.3	0.001	19.5	4.55–83.51	<0.001
**N stage**						
0	Ref.					
1	1.04	0.49–2.20	0.92			
**M stage**						
0	Ref.			Ref.		
1	1.91	0.80–4.56	0.145	3.00	1.16–7.74	0.024
**Tumor length**						
0–5	Ref.					
>5	1.08	0.51–2.27	0.85			
**Gender**						
Male	Ref.					
Female	0.56	0.08–4.01	0.53			
**Smoking**						
No	Ref.			Ref.		
Yes	2.76	0.98–7.78	0.055	4.80	1.56–14.74	0.006
**Drinking**						
No	Ref.					
Yes	0.87	0.38–1.99	0.73			
**Location**						
Upper	Ref.					
Middle	0.82	0.34–2.00	0.66			
Lower	0.88	0.27–2.91	0.84			
**Weight decrease**						
≤ 10%	Ref.					
>10%	1.39	0.64–3.01	0.40			
**RT dose**						
Lower	Ref.					
Higher	0.93	0.47–1.83	0.83			
**Adjuvant chemo**						
No	Ref.					
Yes	0.92	0.44–1.94	0.83			
**pCR**						
No	Ref.			Ref.		
Yes	0.35	0.16–0.74	0.006	0.24	0.10–0.54	0.001

### Complications

Overall, during treatment, 18 (15.2%) patients underwent >10% body weight loss. Anastomotic leakage occurred in 32 patients (36%) and 6 (5.1%) patients died in 30 days following surgery, 2 patients in LD group and 4 patients in HD group. Grade 3 radiation induced esophagitis and pneumonitis were 12.7 and 6.77%, respectively. Treatment-related toxicities were listed in Table 3. The incidences of toxic effects were not significantly different between the two groups, including weight loss, bone marrow suppression, radiation esophagitis, radiation pneumonitis, anastomotic leakage, and 30-days post-operative mortality.

**Table 3:** Adverse events during neoadjuvant chemoradiotherapy and after surgery.

	**LD (*N* = 62)**	**HD (*N* = 56)**	**Total (*N* = 118)**	***P*-value**
**Grade ≥3 events during NCRT**				
Leukopenia	18 (29.0%)	12 (21.4%)	30 (25.4%)	0.72
Thrombocytopenia	5 (8.1%)	7 (12.5%)	12 (10.1%)	0.11
Anemia	6 (9.7%)	6 (10.7%)	12 (10.1%)	0.26
Radiation esophagitis	8 (12.9%)	7 (12.5%)	15 (12.7%)	0.70
Radiation pneumonitis	3 (4.8%)	5 (8.9%)	8 (6.77%)	0.26
Body weight decrease (>10%)	12 (19.4%)	6 (10.7%)	18 (15.2%)	0.19
**Severe complications after surgery**				
Anastomotic leakage	19 (30.6%)	13 (23.2%)	32 (27%)	0.36
Death in 30 days	2 (3.2%)	4 (7.1%)	6 (5.1%)	0.42

## Discussion

Although NCRT is becoming the standard treatment for locally advanced stage EC patients, the optimal radiation dose is still not clear. The results of our study showed that higher radiation dose did not increase the pCR rate significantly, nor improve overall survival. No differences in complications between HD and LD groups were observed in our study. Hence, negative findings were concluded from our study, and no evidence indicates that HD is superior to LD in the neoadjuvant setting for EC patients.

The relationship between radiation dosage and tumor control has been always the research hotspot in radiation oncology. Within certain range, the biological effect was positively correlated with radiation dose. However, the dose-response relationship curve shows “S” type, with a ceiling effect (10, 11). In 2005, after analyzing 177 ESCC patients in Italy, Grandinetti et al. found that induction chemoradiotherapy with 50 Gy RT led to an increase of pCR rate and improved OS, comparing to 30 Gy RT (12). Then, one systematic analysis of 26 trials also indicated that dose escalation was associated with higher pCR rate within 20–60 Gy, concurrent with 5FU, cisplatin based chemotherapy regimen (13). However, the lowest doses in these studies were <40 Gy, whether the positive association between radiation dose and pCR rate also exist among 40–50.4 Gy is unclear, the level of which might fall in the shoulder area of “S” type. In fact, another large randomized trial (RTOG9405) which used high-dose (64.8 Gy) radiotherapy failed to improve overall survival as well as local control, comparing to standard dose (50.4 Gy), in the context of definitive concurrent CRT for esophageal cancer (14), which seems to exist in other kinds of cancer (15, 16). Possibly, for sensitive cancer cells, a dose of 40–45 Gy irradiation can effectively eliminate the tumor cells. However, for those primary radiation-resistant cells, increased dose does not bring better outcomes, or only in a slight degree. Indeed, in the present study, the pCR rate in HD group was higher than in LD group, but the result was statistically non-significant, and both DFS and OS were not improved with higher irradiation dose. In addition, concurrent chemotherapy might also weaken the positive association between radiation dose and tumor control (17, 18).

The purpose of neoadjuvant therapy is to increase R0 resection rate and reduce local recurrence, thus to improve long-term overall survival. Recently, in both CROSS and NEOCRTEC5010 studies, lower doses (41.4 Gy/23F or 40 Gy/20F) of radiotherapy were employed, which showed high efficacy and brought positive results, with pCR rate >40%, R0 rate >90%, and only 5.2% recurrence rate in the irradiation field, indicating that lower irradiation of 40 Gy can also kill tumor cells effectively (6, 7). In fact, the use of NRCT does not need to eradicate all the tumor cells; pathological major response (pMR) can be also accepted, since the subsequent surgery will further remove the tumor. Therefore, relatively lower dose radiation therapy can achieve the goal to control the micrometastatic lesions and bring survival benefits. Recently, several studies using National Cancer Data Base reported different results concerning the impact of radiation dose on the survival or pCR rate in esophageal cancer patients receiving neoadjuvant chemoradiation therapy (19–22); three studies found irradiation dose had no impact on survival of before or after propensity matching, and one study even concluded that 41.4 Gy is associated with superior overall survival, compared to 50.4 Gy (19). Another two retrospective studies also revealed that higher neoadjuvant radiation dose did not enhance pCR rate in esophageal cancer (23, 24).

The side effects of NCRT are another important issue to consider, especially complications after esophagectomy. Most randomized studies showed that NCRT did not increase the risk of post-operative events, including anastomotic leakage, mediastinitis, pulmonary complications, and 30-days post-operative death (6, 7, 25), even in patients with higher dose (50.4 Gy) irradiation (9), which was verified by meta-analysis (4). Although the overall incidence of anastomotic leakage was relatively higher in our study than those reported in previous studies (6, 7, 9), no significant differences of post-operative events were observed between HD and LD groups, and the most common cause for deaths in 30 days after surgery was pulmonary complications.

Currently, NCCN guideline recommends 41.1–50.4 Gy for NCRT in EC patients (26). In practice, according to the results from a national survey of ASTRO members, 50.4 Gy was the most common dose used in NCRT of esophageal cancer in North American (27). However, lower radiation doses 41.4 or 40 Gy were used in recent large clinical trials, and promising results were obtained, which gave radiation oncologists other choices. Notably, personalized radiotherapy scheme should be made for individual patients in clinical practice. For those potentially resectable locally advanced stage EC patients, some still not suitable for R0 resection even after NCRT, higher dose of 50.4 Gy might be more appropriate, which is standard dose for definitive chemotherapy. On the other hand, for those early operable EC patients at diagnosis, lower dose of 40–45 Gy might be more appropriate, since it is reported that NCRT might increase post-operative mortality in patients with early-stage EC (28).

Several limitations have to be admitted in our study. Firstly, the retrospective nature was prone to recall bias, selection bias, and information loss, which might result in imbalanced baseline characteristics between groups. Secondly, a small sample size decreased the statistical power to detect a non-obvious difference, and also limited further subgroup analyses as well as propensity score-matched analysis. Thirdly, 5-years overall survival cannot be obtained by reason of short-time follow-up. In consideration of these limitations before, a prospective randomized trial has been designed to explore the optimal dose in the neoadjuvant setting for EC patients in our institution (NCT03381651), which is now recruiting patients.

## Conclusion

In ESCC patients treated with NCRT followed by surgery, higher radiation dose does not increase pCR rate or improve overall survival, comparing to lower dose, although no differences in the side-effects were observed between the two groups. Therefore, lower radiation dose, including 40 Gy/20F, 41.4 Gy/23F, or 45 Gy/25F, might be a preferable time-dose fraction scheme, which will decrease medical cost and shorten the duration of hospitalization. Our finding needs to be further validated by randomized trials.

## Data Availability Statement

All datasets generated for this study are included in the article/supplementary material.

## Ethics Statement

The studies involving human participants were reviewed and approved by the ethics committee of Zhejiang Cancer Hospital. The patients/participants provided their written informed consent to participate in this study.

## Author Contributions

QC and GQ conceived and drafted the study. YY, XX, XZ, WB, DZ, FG, QC, and GQ treated and followed all the patients and collected all the data. YY, XX, and XZ analyzed and interpreted the data. All authors commented on drafts of the paper and approved the final manuscript.

## Conflict of Interest

The authors declare that the research was conducted in the absence of any commercial or financial relationships that could be construed as a potential conflict of interest.
